# Small-scale variation of ammonia oxidisers within intertidal sediments dominated by ammonia-oxidising bacteria *Nitrosomonas sp*. *amoA* genes and transcripts

**DOI:** 10.1038/s41598-017-13583-x

**Published:** 2017-10-16

**Authors:** Aoife M. Duff, Li-Mei Zhang, Cindy J. Smith

**Affiliations:** 1 Microbiology, School of Natural Sciences, NUI Galway, Galway, Ireland; 2 State Key Laboratory of Urban and Regional Ecology, Research Centre for Eco-Environmental Science, Chinese Academy of Sciences, 18 Shuangqing Rd., Haidan Beijing, 100085 P.R. China; 3 Present Address: Infrastructure and Environment, School of Engineering, University of Glasgow, Glasgow, United Kingdom

## Abstract

While numerous studies have investigated the abundance of ammonia oxidising bacteria and archaea (AOB/AOA) via the ammonia monooxygenase gene *amoA*, less is known about their small-scale variation and if *amoA* gene abundance equates to activity. Here we present a spatial and temporal study of ammonia oxidation in two small intertidal bays, Rusheen and Clew bay, Ireland. Potential Nitrification Rate (PNR) was ten-fold higher in Rusheen bay (Clew: 0.27 ± SD 0.55; Rusheen: 2.46 ± SD 3.4 NO_2_^−^ µg^−1^ g^−1^ day^−1^, P < 0.001) than in Clew bay but *amoA* gene abundances were similar between bays, and comparable to those in other coastal ecosystems. Within bays AOB genes increased towards the muddy sediments and were positively correlated with PNR and pH. Less spatial variation was observed in AOA abundances which nevertheless positively correlated with pH and temperature and negatively with salinity and ammonia. Transcriptionally active AOB and AOA were quantified from all sites in Rusheen bay, February 2014, following the general trends observed at DNA level. AOB phylotypes predominantly from the known *Nitrosomonas* group were distributed across the bay, while *Nitrosomonas* group B phylotypes were absent from low salinity sites. AOA genes and transcripts were primarily affiliated with *Thaumarchaeota* group I.1a.

## Introduction

Nitrogen is a limited element for primary productivity. While nitrate-limited environments suffer from low primary productivity, excess nitrate in the environment is problematic as it leads to eutrophication and hypoxia. Coastal ecosystems are particularly vulnerable to excess nitrogen loads as they are transition regions between terrestrial and marine environments, where land, streams, groundwater and rivers meet the sea. However, microbial processes within coastal ecosystems mediate the nutrient load entering coastal waters via the various steps of the nitrogen cycle, including nitrification, denitrification, dissimilatory nitrate reduction to ammonia and anammox^1–3^. In fact, it is estimated that greater than 50% of anthropogenic dissolved inorganic nitrogen (DIN) inputs to coastal ecosystems are removed by microbial transformations of the nitrogen cycle^4^.

Nitrification, the oxidation of ammonia to nitrite and then nitrate, is a central process of the nitrogen cycle. Through coupling with denitrification (the stepwise reduction of nitrate to di-nitrogen gas), it plays an important role in mitigating associated risks of increased DIN^5^. Traditional understanding of nitrification is a two-step process whereby ammonia oxidising bacteria (AOB) or archaea (AOA) oxidize ammonia to nitrite. Nitrite is then oxidised to nitrate by nitrite oxidising bacteria. The recent discovery of complete ammonia oxidation to nitrate (comammox) by organisms within the genus *Nitrospira* ^6^ adds a new dynamic to current understanding.

In two-step nitrification, ammonia oxidation, the first and rate-limiting step, is conducted by AOA and AOB. The ammonia monooxygenase (AMO) enzyme converts ammonia to nitrite^7^ and is encoded by the *amo* gene^8^. *amoA* is frequently used as a functional marker to detect, quantify and identify AOA and AOB in the environment^9^. Using this functional gene marker approach, the abundance of AOA and AOB has been extensively surveyed in a range of coastal and estuarine environments to infer the distribution of AOA and AOB^10–17^. While AOA and AOB are both present, niche differentiation is often apparent^18–20^ and abiotic factors such as ammonia concentration, oxygen, temperature, pH and salinity have been shown to influence abundance and distribution^21–25^. Within coastal sediments, in some cases AOB have been reported as more abundant^13,26,27^ whilst other studies report AOA as dominant^28,29^. Generally a trend of greater AOA at low ammonia^18,30^ verses AOB at high ammonia concentrations is evident^31–34^. What is not understood is if gene abundance equates to activity. For this, the detection and quantification of *amoA* mRNA transcripts would be more informative than DNA alone^35^.

Further, to-date, the majority of studies in coastal sediments have been conducted over large spatial scales (e.g. 5 to 330 km^26,36–39^). Nitrifier dynamics over smaller spatial scales and active ammonia oxidisers (AO) within intertidal sediments are less well understood. It has been shown that within saltmarshes significant differences in AO diversity occur over local scales (3 cm to 1 km), that are not observed at larger regional (1 to 100 km) or continental (up to 12,500 km apart) spatial scales^40^. Intertidal bays are dynamic ecosystems that vary on both temporal and spatial scales largely driven by tidal variations. Further within a bay, differences in sediment characteristics occur with organic matter accumulating in low energy areas resulting in muddy sediments verses sandier sediments of the high energy open bay. In addition, any freshwater inputs from rivers, streams and groundwater alter salinity and nutrient concentrations. Thus we hypothesise that such small-scale variation in environmental parameters is likely to drive differences in nitrification activity, diversity and abundances. To this end, we investigated nitrification across a range of sediment types and salinities within and between two small intertidal bays (~1 km^2^) on the west coast of Ireland over an annual period. Contrasting bays, one located in a rural (Clew bay) and the other in an urban setting (Rusheen bay; approximately 100 km apart) were selected. Two bays were selected to compare and contrast nitrifier abundance and activity across similar environmental gradients (TOC, salinity, sediment type, nutrients, pH) within each to elucidate whether observed differences are greater within or between bays for a given set of environmental drivers. The objectives of the study were to monitor nitrification potential in addition to AOA and AOB *amoA* gene abundances within and between bays over an annual cycle. Furthermore, we aimed to quantify and identify active ammonia oxidisers *in situ* by targeting *amoA* gene transcripts within a single-bay and time-point. Intertidal bays are highly complex and dynamic ecosystems; we therefore hypothesised that nitrifier activity and *amoA* gene abundances would vary spatially and temporally within and between bays due to fluctuating environmental parameters. Furthermore, we postulated that AOB would be more abundant than AOA due to elevated ammonia concentrations; and that AOA and AOB diversity would vary with changing environmental parameters within intertidal bays.

## Results

### Site description

**Figure 1:**
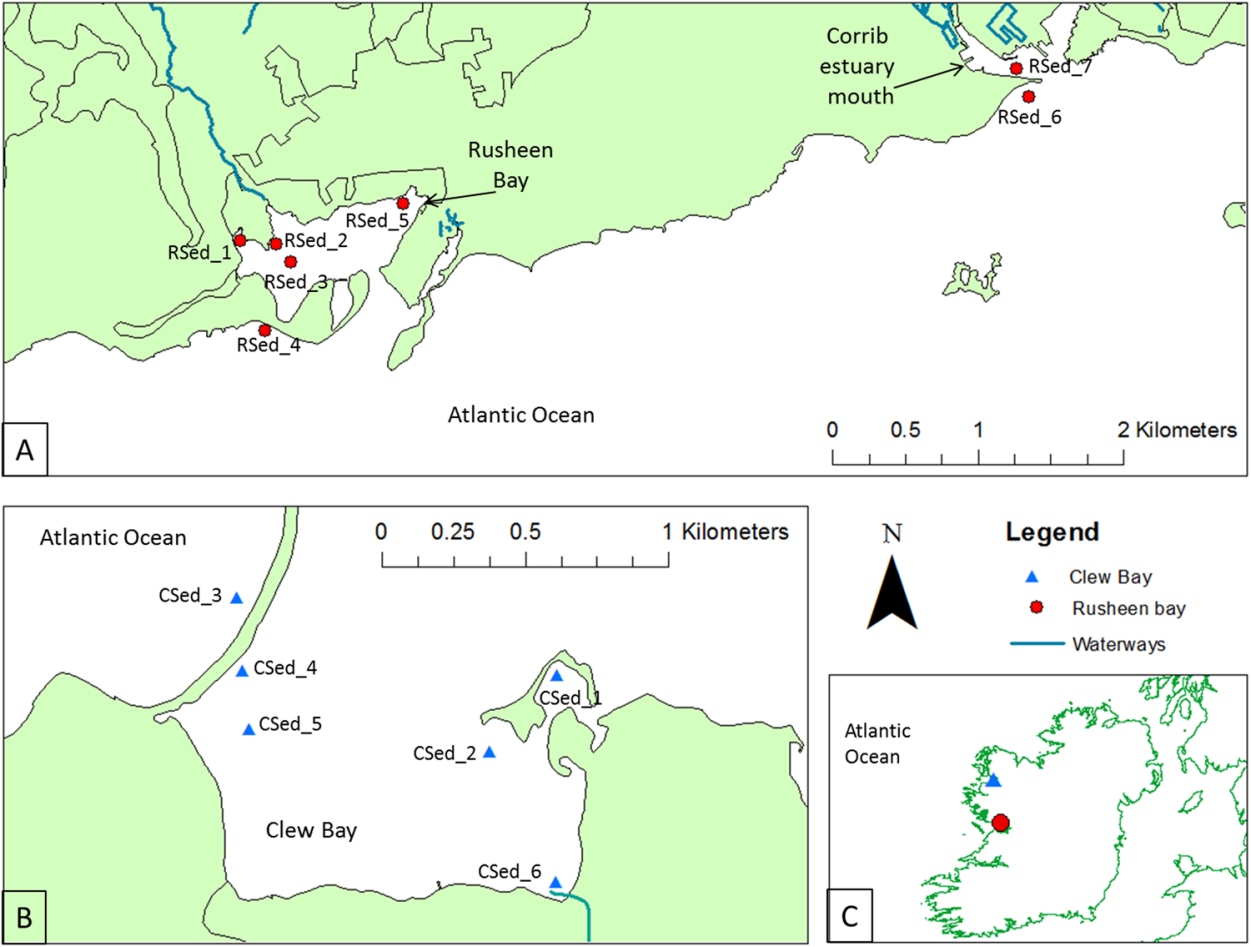
Location of sampling sites within (**A**) Rusheen Bay (sites RSed_1 to RSed_5) and at the Corrib estuary mouth (RSed_6 & 7), (**B**) Clew Bay (sites CSed_1 to CSed_6) and (**C**) Location of Rusheen and Clew Bay on the West Atlantic coast of Ireland. Map was drawn using ArcGis V10 (www.arcgis.com).

Sites representing sandy (RSed_3, RSed_4, RSed_6, RSed_7, CSed_2, CSed_3 and CSed_4) to muddy (RSed_1, RSed_2, RSed_5, CSed_1, CSed_5 and CSed_6) gradients in addition to low salinity (<10 psu) sites (RSed_6, RSed_7 and CSed_6) in each bay, were sampled every three months from May (2013) to February (2014) (Fig. 1). Physical-chemical parameters are reported in Supplementary Table S1A and B. pH ranged between 6.65–8.43, increasing from May to November (One-way ANOVA; P < 0.05). NH_4_^+^ concentrations were higher in Rusheen (range: 0–1166.2, average: 283.55 ± SD 38.68 µM^−1^ g^−1^ fs^−1^) and lower in Clew bay in April and November, (range: 0–269.11 µM^−1^ g^−1^ fs^−1^ average, 63.76 ± SD 16.99 µM ^−1^ g^−1^ fs^−1^ NH_4_^+^, One-way ANOVA P < 0.05).

### Spatial and temporal variation in potential nitrification rates and AOA/AOB *amoA* gene abundances

**Figure 2:**
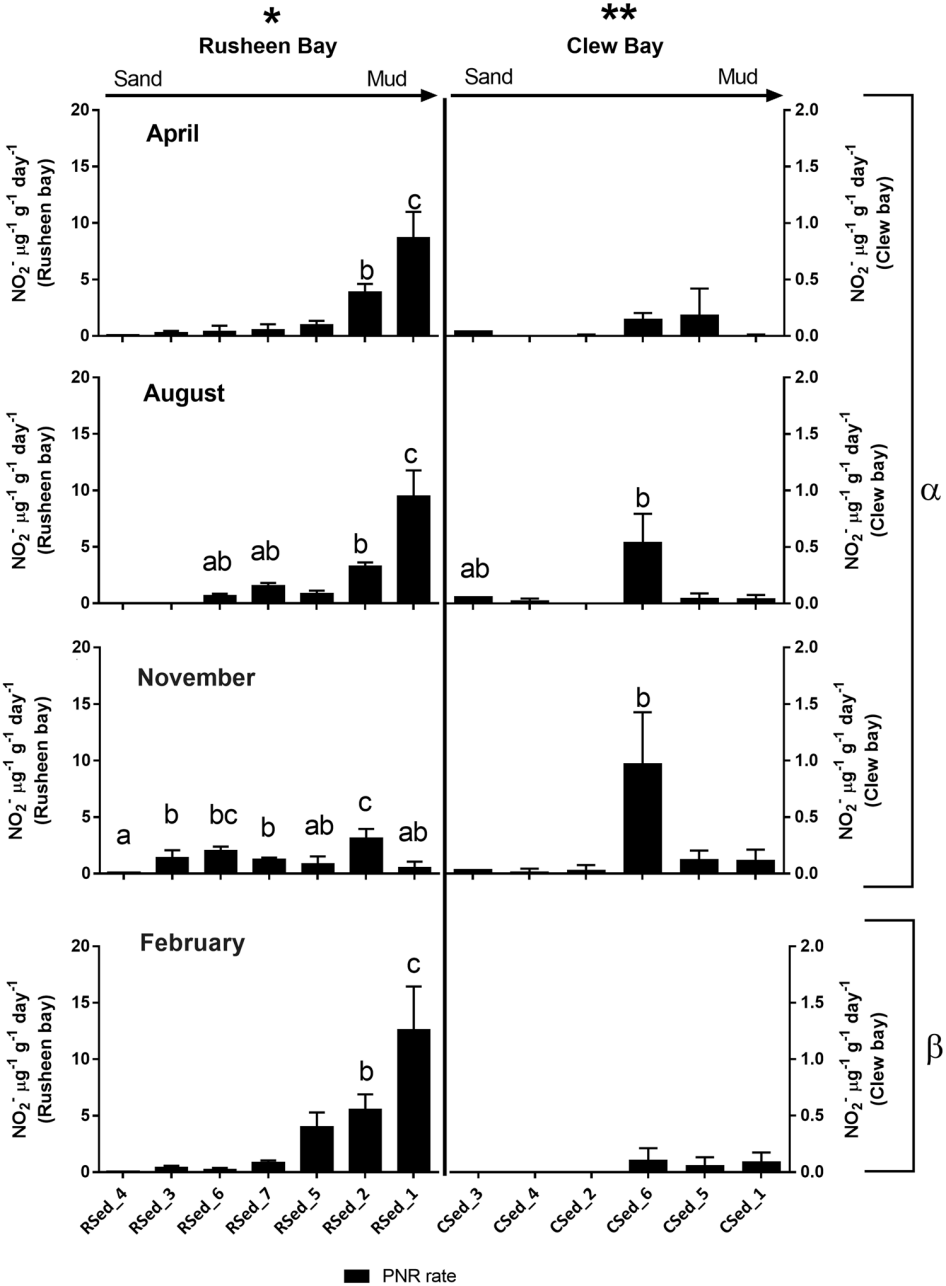
Spatial and temporal variation in Potential Nitrification Rates (PNR) (PNR NO_2_^−^ µg^−1^ g^−1^ day^−1^) in Rusheen and Clew bay sediments (mean n = 3, standard deviation indicated by error bars). PNR in Rusheen bay are located on the left y-axis, Clew bay is on the right y-axis. Sites are arranged in order of sandy sediments to muddy sediments. Four time points include rates measured in April, August, November 2013 and February 2014. Letters indicate significant differences between sites within a bay (One-way ANOVA, P < 0.05), Greek letters indicate significant differences between time points and the asterisk symbols indicate significant differences between bays (Three-way ANOVA, P < 0.05).

PNR in Rusheen (range: 0 to 12.65 µg^−1^ g^−1^ d^−1^) was up to a log-fold greater than Clew bay (range: 0 to 2.14 µg^−1^ g^−1^ d^−1^; Fig. 2; Three-way ANOVA P < 0.001). Within Rusheen bay, PNR varied spatially increasing towards the muddy-sediments (range: 0.06 to 12.65 µg^−1^ g^−1^ d^−1^; P < 0.05), in all months except November. Within Clew bay PNR did not vary spatially with the exception of the low salinity site (CSed_6) which was significantly higher than all other sites in August (0.54 µg^−1^ g^−1^ d^−1^) and November (0.97 µg^−1^ g^−1^ d^−1^; One-way ANOVA P < 0.05). Temporally, within each bay, there was no difference in PNR between April, August and November; however, PNR was higher in Rusheen bay, but lower in Clew bay in February than all other time-points (Fig. 2; Three-way ANOVA; P < 0.05).

**Figure 3:**
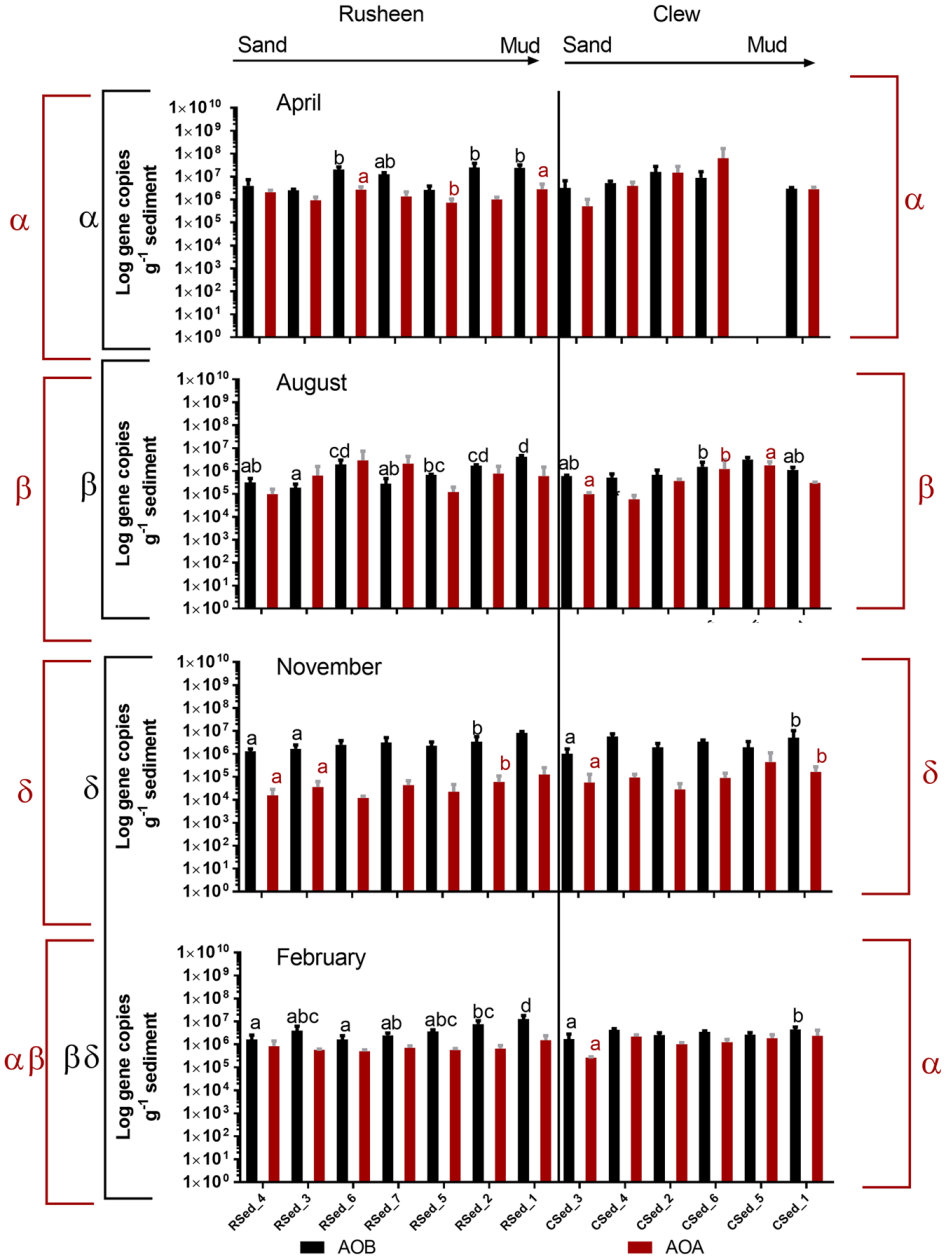
Spatial and temporal variation in AOA (red) and AOB (black) *amoA* gene abundances in Rusheen and Clew bay sediments. Significant differences within a bay are indicated by letters above the site (One-way ANOVA, P < 0.05). Significant differences between time-points are indicated by Greek symbols (Two-way ANOVA, P < 0.05).

AOB *amoA* gene abundances ranged from 1.89 × 10^5^ to 2.5 × 10^7^ (mean ± SD 3.81 × 10^6^ ± 1.5 × 10^7^) copies per gram of sediment (Fig. 3). In contrast to the lower PNR in Clew bay (Three-way ANOVA; P < 0.001) there was no difference in AOB *amoA* gene abundances between bays (Three-way ANOVA; P = 0.180). Within each bay, AOB *amoA* gene generally increased towards the muddy sites (One-way ANOVA; P < 0.05, Fig. 3). Temporally, within Rusheen bay, AOB *amoA* gene abundances were similar in the winter months (November and February) but were significantly different between April and August (One-way ANOVA; P < 0.05). No temporal change was observed in Clew bay. AOA *amoA* gene abundances ranged from 1.21 × 10^4^ to 6.29 × 10^7^ (mean ± SD 2.27 × 10^6^ ± 1.14 × 10^6^) copies per gram of sediment (Fig. 3) with AOA *amoA* gene abundances statistically higher in Clew bay (Three-way ANOVA; P < 0.05). AOA gene abundances were generally the same within a bay, with any significant increase observed at muddy sediment sites only (One-way ANOVA; P < 0.05).

AOB gene abundances correlated positively with PNR, *in situ* TOC and *in situ* pH (r = 0.509, P < 0.001; r = 0.434, P < 0.001; r = 0.315 P < 0.05); and negatively with temperature and NH_4_^+^ concentration (r = 0.459 P < 0.001; r = 0.404 P < 0.001). AOA gene abundances positively correlated with *in situ* pH and temperature (r = 0.69 P < 0.001; r = 0.242 P < 0.05) and negatively with *in situ* salinity and NH_4_^+^ concentration (r = −0.245, P < 0.05; r = −0.438, P < 0.001). PNR positively correlated with *in situ* TOC and sediment salinity (r = 0.223, P < 0.05; r = 0.265, P < 0.05; Supplementary Table S2).

### Abundance and community shift of *amoA* transcripts across Rusheen bay

**Figure 4:**
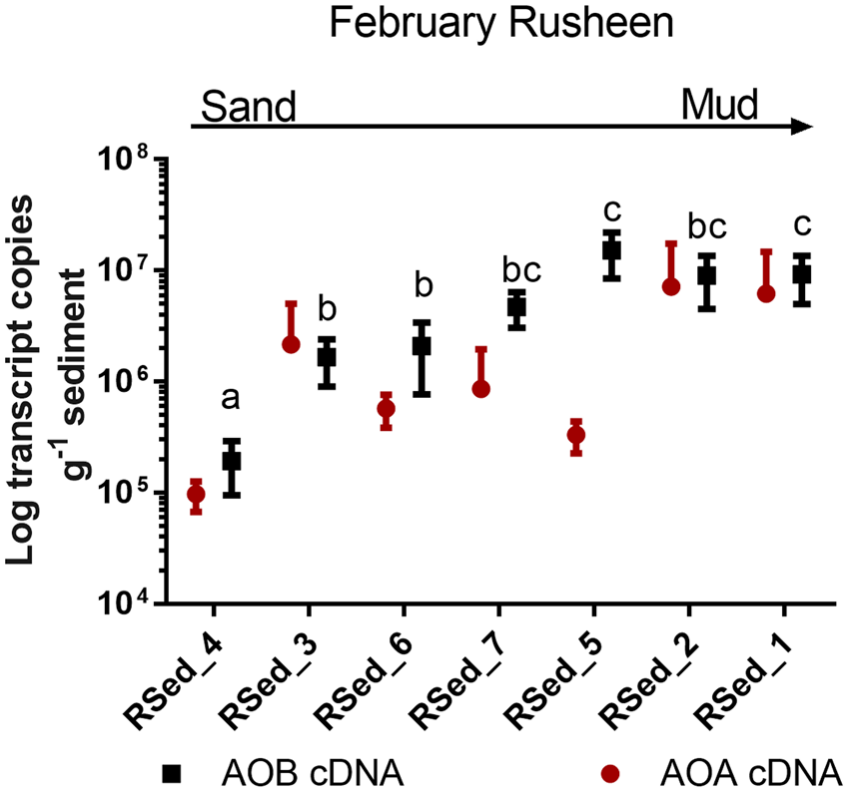
Log_10_ AOA and AOB transcript numbers g^−1^ sediment for February 2014 in Rusheen bay. AOB numbers are indicated by the black squares while AOA are indicated by the red circle. The black letters represent the significant differences between sites across Rusheen bay for AOB transcripts (One-way ANOVA, P < 0.001).

Rusheen bay, February 2014 was selected to further investigate the diversity and abundance of *amoA* DNA (total) and mRNA (active) from AOA and AOB based on the overall higher rates of PNR in Rusheen than Clew bay. Only February sediments were targeted as we wanted to use fresh sediments to investigate transcript activity. AOB *amoA* transcripts across the bay ranged from 1.93 × 10^5^–1.52 × 10^7^ per gram of sediment with an average 6.02 × 10^6^ ± SD 2.77 × 10^6^ and were greater than AOA *amoA* transcripts at all sites except for Rsed_3 (9.7 × 10^4^ − 6.2 × 10^6^ per gram of sediment, average 2.49 × 10^6^ ± SD 3.31 × 10^6^; Fig. 4). AOB *amoA* transcripts increased from the sandier to muddier sediments (One-way ANOVA, P < 0.001). AOA *amoA* transcripts were quantified from all sites, but no spatial difference was observed (One-way ANOVA, P = 0.126). AOB *amoA* transcripts correlated positively with PNR (r = 0.868, P < 0.001), while AOA did not; in addition, AOB *amo*A transcripts and *in situ* nitrate concentrations were significantly correlated (r = 0.444, P < 0.05) (Spearman rank correlations; Supplementary Table S3).

**Figure 5:**
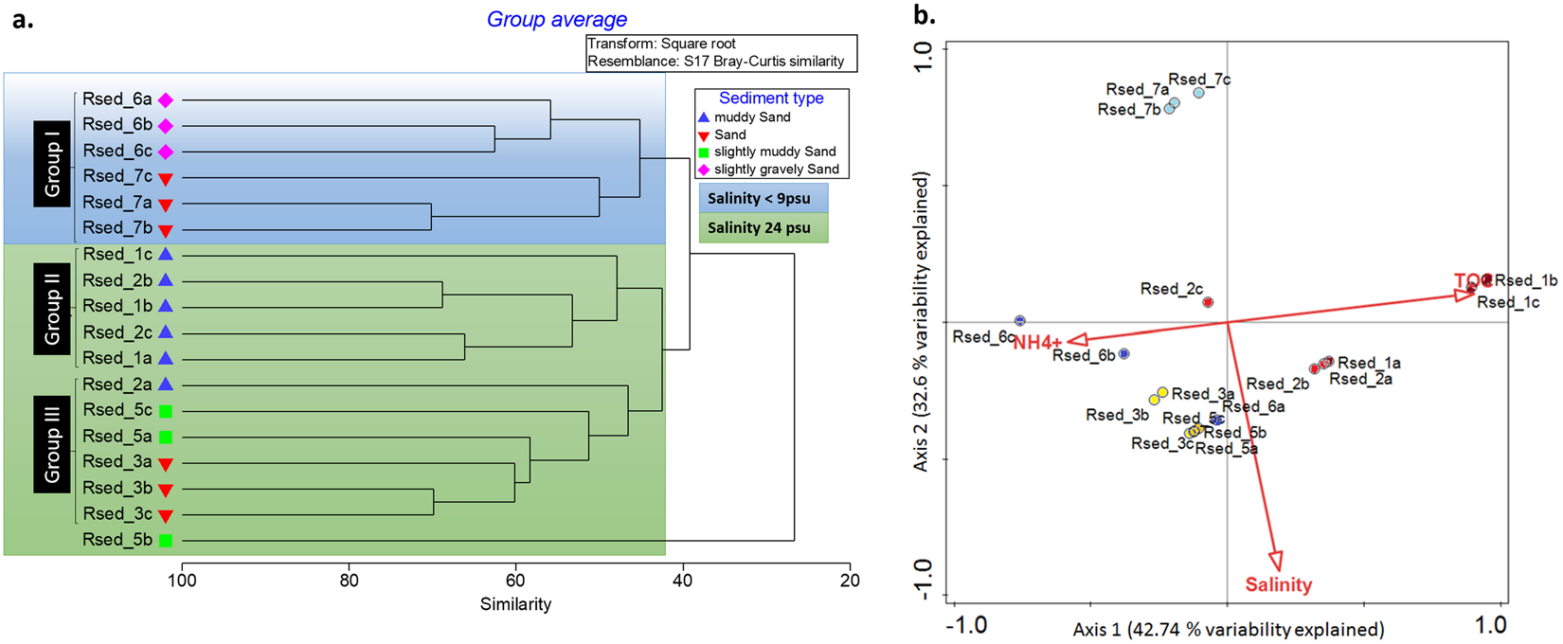
AOB *amoA* transcript community analysis (**a**) Cluster dendrogram representing T-RFLP profiles of AOB *amoA* transcripts from Rusheen bay sediments February 2014. Scale bar: Bray-Curtis similarity coefficient. Clusters highlighted in blue and green indicate salinity below 9 psu and equal to 24 psu respectively. Shapes represent different sediment types from muddy sand to sand. (**b**) Canonical correspondence analysis illustrates the separation of AOB cDNA *amoA* gene TRFs based on physical/chemical parameters measured. The different colours represent an individual site. The most significant parameters are represented by the labelled vectors. The abbreviations PNR and TOC represent the potential nitrification rates and total organic carbon respectively.

To determine if different active AOB *amoA* phylotypes were present across the bay, T-RFLP was conducted. Site RSed_4, the coarse sand site, was excluded due to low PCR yield. Three groups of AOB *amoA* cDNA phylotypes, at 45% similarity, were present (Fig. 5a) – group I the low salinity sites, Rsed_6 and 7 (ANOSIM r = 0.815, P = 0.1); group II the muddy sites, RSed_1 and 2 (ANOSIM, r = 0.37, P = 0.2) and group III the sandy sites, RSed_3 and RSed_5 (ANOSIM r = 0.333, P = 0.1). Furthermore, CCA showed that a combination of ammonium (NH_4_^+^; f = 1.6, P = 0.024) and TOC (f = 2.05, P = 0.004) explained 42.74% of variation of AOB phylotypes present (Fig. 5b). After which, salinity (f = 1.73, P = 0.008) was the next significant variable explaining 32.6% of the distribution of active AOB phylotypes across the bay.

### *amoA* gene and transcript phylogeny of ammonia oxidisers within Rusheen bay sediments

Based on the cluster dendrogram (Fig. 5a) sites RSed_1, 5, 6 & 7 were selected for *amoA* AOA/AOB gene and transcript sequencing. In general, DNA PCR amplicons were too weak for Illumina MiSeq analysis so a clone library approach was used. In total, 68 AOB DNA and 197 cDNA *amoA* sequences and 61 AOA DNA and 32 cDNA sequences, correctly translating to protein were recovered. From these, thirty-six DNA and 41 cDNA AOB OTUs and 19 DNA and 8 cDNA AOA OTUs at 97%^41^ similarity were identified (Supplementary Table S4). *amoA* AOB transcripts were retrieved from all sites, but *amoA* AOA transcripts were only recovered from the sandy sediment sites RSed_5 (high salinity) and RSed_6 (low salinity).

**Figure 6:**
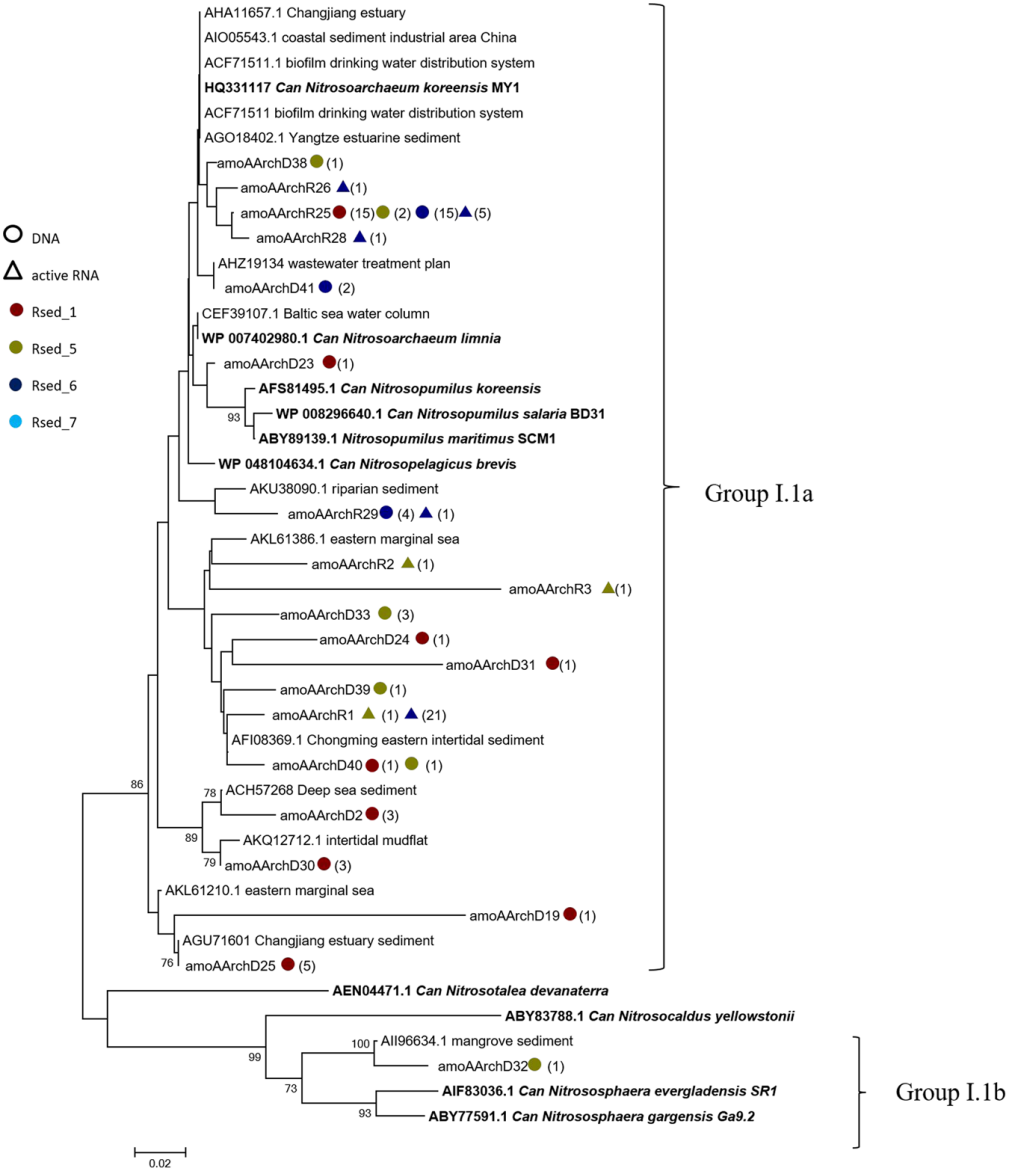
Neighbour-joining phylogenetic tree of AOA *amoA* genes at protein level (≥ 97% similarity). Bootstrap values shown are greater than 70% of 1000 are shown near nodes. Accession numbers from GenBank are shown from other studies and isolates. Circles indicate DNA sequences, triangles indicate cDNA sequences. The numbers in brackets indicate the number of sequences recovered from that site. The colours maroon, yellow, blue and aqua signify different sampling sites RSed_1, RSed_5, RSed_6 and RSed_7 respectively.

Archaeal *amoA* grouped into *Thaumarchaeota* Group I.1a, and Group I.1b. (Fig. 6). The majority of AOA fell into *Thaumarchaeota* group I.1a, with a single OTU from RSed_5 falling into group I.1b. The sequences affiliating with group I.1b shared high identity to *Can*. *Nitrososphaera evergladensis* and *gargensis* and the sequence from mangrove sediments. One cluster, closely related to *Can*. *Nitrosoarchaeum koreensis MY1*, contained the majority of OTUs recovered from all sites. A single DNA phylotype (amoAArchD23) from RSed_1, the high salinity muddy sediment site, clustered with *Nitrosopumilus maritimus*, and two candidate *Nitrosopumilus sp*. The remaining sequences in Group I.1a clustered with environmental clones generally from similar coastal environments.

**Figure 7:**
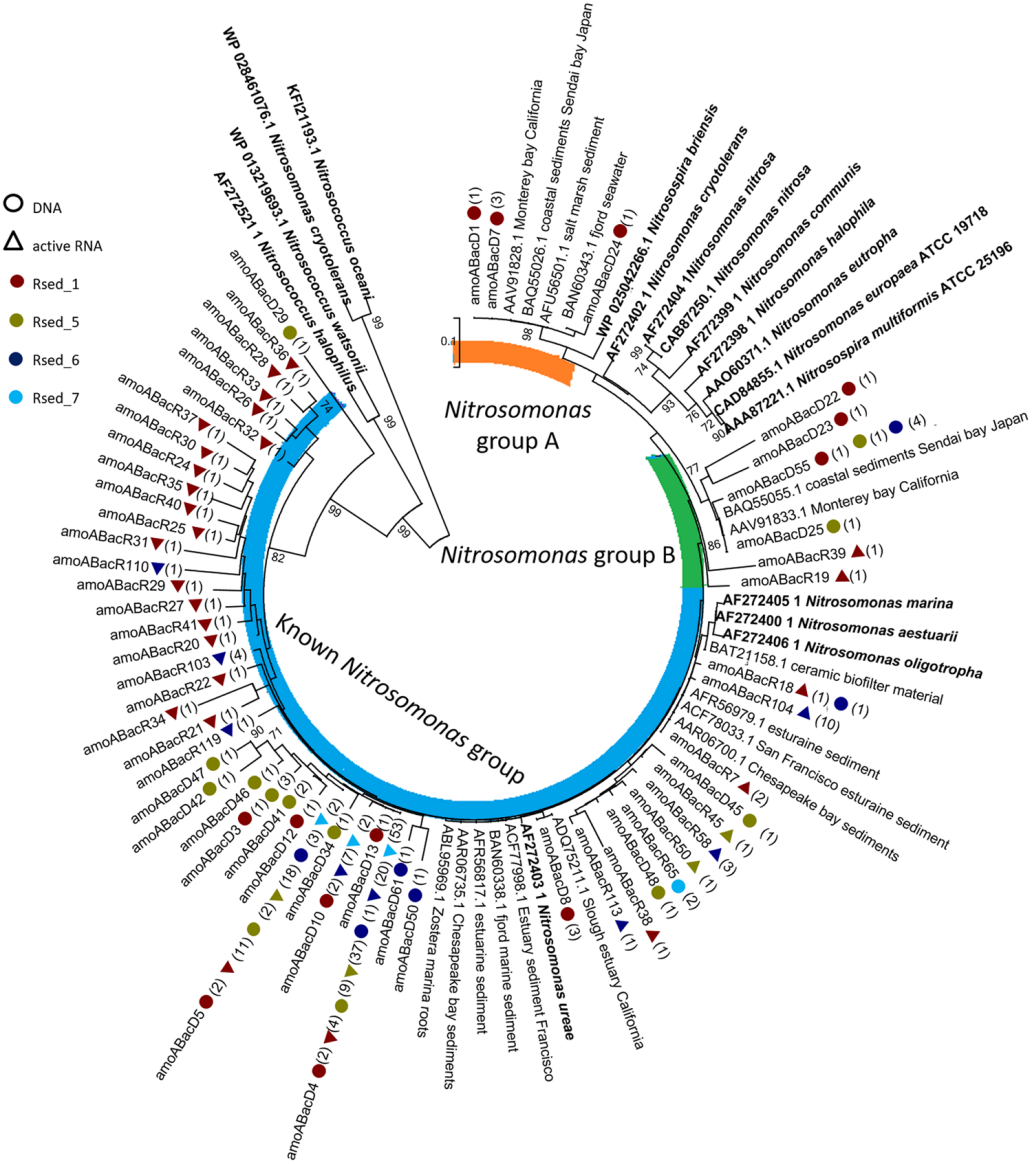
Neighbour-joining phylogenetic tree of AOB *amo*A genes at protein level (≥ 97% similarity). Bootstrap values shown are greater than 70% of 1000 are shown near nodes. Accession numbers from GenBank are shown from other studies and isolates. Circles indicate DNA sequences, triangles specify cDNA sequences. The numbers in brackets indicate the number of sequences recovered from that site. The colours maroon, yellow, blue and aqua signify the sampling sites RSed_1, RSed_5, RSed_6 and RSed_7, respectively.

All AOB fell into the *Nitrosomonas* lineage, which was further divided into three clusters; the known *Nitrosomonas* group, and *Nitrosomonas* group A and B (Fig. 7), corresponding to a similar classification as used in O’Mullan and Ward, 2005. The known *Nitrosomonas* group accounted for 93.4% of sequences, including the dominant OTUs found at all sites (*amoA*BacD4 and D5). These sequences were affiliated with isolates and enrichments *N*. *aestuarii*, *N*. *ureae*, *N*. *oligotropha* and *N*. *marina*. *Nitrosomonas* group A cluster contains OTUs from the high salinity muddy sediment site, RSed_1 only. These were highly similar to *amoA* sequences from other coastal ecosystems distributed globally. The *Nitrosomonas* group B cluster contained sequences from three sites (RSed_1, 5 and 6, muddy and sandy sediments) and absent from Rsed_7, the lowest salinity site (<1 psu). These clustered with *amoA* phylotypes from similar high salinity coastal ecosystems.

To identify AOB cDNA TRFs, *in silico* TRFs were generated from *amoA* gene sequences and compared to observed TRFs (Table 1). TRFs representing the two dominant *amoA* gene sequences (*amoA*BacD4 and D5, Fig. 7) from the known *Nitrosomonas* group, were present at all sites. In addition, TRFs equating to two other phylotypes within the known *Nitrosomonas* group (*amoA*BacR42 and 47) were ubiquitously present. TRFs representing *Nitrosomonas* group A (*amoA*BacD1, D7 & D24) were distributed across the bay. However, TRF’s representing the *Nitrosomonas* group B, were present at the high salinity sites only.

**Table 1:** Distribution of key AOB phylotypes across Rusheen bay identified from cDNA T-RFLP profiles.

Enzymes	Cluster	*Cfo 1*	*TaqI*	*AluI*	*Cfo 1*	*TaqI*	*AluI*
Phylotype		*in silico*	*in silico* TRF	*in silico* TRF	Actual TRF	Actual TRF	Actual TRF
amoABacD4	Known *Nitrosomonas* group	431	431	431	493.56	493.62	493.6
amoABacD5	Known *Nitrosomonas* group	431	46	431	493.56	41.29–46.55	493.6
amoABacD42	Known *Nitrosomonas* group	431	431	256	493.56	493.62	273.18–274.73
amoABacD47	Known *Nitrosomonas* group	249	46	431	206.08 – 283.44	41.29–46.55	493.6
amoABacD1	*Nitrosomonas* group A	66	0	0	64.3–67.72	0	0
amoABacD7	*Nitrosomonas* group A	66	29	223	64.3–67.72	0	220.61–228.22
amoABacD24	*Nitrosomonas* group A	66	292	343	64.3–67.72	284.32–287.43	351.65–356.14
amoABacD22	*Nitrosomonas* group B		281			275.92–279.66	
amoABacD23	*Nitrosomonas* group B		281			275.92–279.66	
amoABacD25	*Nitrosomonas* group B		281	223		275.92–279.66	220.61–228.22

Site legend: Rsed_1 Rsed_2 Rsed_3 Rsed_5 Rsed_6 Rsed_7.

## Discussion

Spatial variation in nitrification and drivers of activity, abundance and distribution of AO were investigated within and between intertidal bays. Rusheen bay (0–12.6 µg^−1^ g^−1^d^−1^) had higher nitrifier activity than Clew bay (0–0.972 µg^−1^ g^−1^d^−1^). The reason for the difference in activity between the bays was not revealed in this study. Rusheen bay PNRs were slightly higher compared to similar environments e.g. Western English Channel (1.344–9.408 µg^−1^ml^−1^d^−1^), salt marsh sediments, Skidaway island (0–7.728 µg ^−1^g^−1^d^−1^) but lower than the hypernutrified Colne estuary, UK (126–546 µg ^−1^ g^−1^d^−1^)^27,42,43^. Small scale spatial variation in PNR was observed with rates increasing towards the muddy sediments (0.064–12.648 µg^−1^ g^−1^ d^−1^). This is consistent with Zheng *et al*., (2014) who also reported highest nitrification in muddy sediments and lowest in sandy sediments^37^. In contrast, no difference in PNR was reported between sandy and muddy sediment types in deep-sea sediments^44^. In Rusheen bay, intermediate salinities (16.66 and 24 psu) were recorded at sites RSed_1–5, August 2013 and February 2014 respectively. Both time-points had higher nitrification rates compared to the higher salinity (~32 psu) time-points (April and November 2013). While, in general there was no difference in PNR activity across Clew Bay, when significant increases were observed it was always associated with the low salinity site (CSed_6; 0–0.23 psu). Similarly, studies from a number of other temperate estuaries report highest nitrification at the intermediate salinities (5 to 20 psu)^27,45,46^.

AOB at DNA level, were 1 to 2 orders of magnitude greater than AOA *amoA* at all four-time points in both bays. *amoA* gene copies per cell in AOB range from 1 to 3^47^ while a single copy is present in AOA^48^. Adjusting AOB *amoA* gene abundances for the maximum *amoA* gene copies per cell they still outnumbered AOA. Despite the variation in PNR between bays, AOB *amoA* gene abundances were not different between bays. Indeed AOB *amoA* gene abundances from both bays in this study were similar in range to other coastal bay and intertidal sediments where AOB were also more abundant than AOA^27,37,39,49^. In contrast other water column and submerged coastal and estuarine sediment studies have reported AOA as more abundant^26,28,38,50–52^. Further there was a difference in AOA *amoA* gene abundance between the bays in this study, however they were still within the range of those reported in other coastal sediments^14,39^.

AOB gene abundances correlated significantly with PNR in both bays, and thus also shared the general trend of increasing in abundance towards muddy sediments. Further AOB abundance was positively correlated with TOC, pH and negatively with NH_4_^+^ concentration (Supplementary Table S2). Similar observations were also seen in the Yangtze estuary and bioturbated sediments^24,37^. The large seasonal variation in TOC (Supplementary Table S1a and b) may have contributed to an increase in ammonia via increased mineralisation. This potentially had a knock on effect on nitrification, explaining the correlation of AOB gene abundances, transcripts and PNR with TOC (Supplementary Tables S2 and S3). No correlation of AOB abundance with salinity was seen. AOA *amoA* gene abundances also tended to show spatial change across the bays increasing towards the muddy sediment although the trend was less distinct than for AOB. AOA gene abundance was correlated with both NH_4_^+^ and salinity. Similar observation have been shown in other coastal sediments and AOA are thought to play a more significant role in low salinity ecosystems^26,37,53^.

Both AOB and AOA *amoA* transcripts were quantified *in situ* from all sites in Rusheen bay, February 2014, indicating the presence of transcriptionally active AOB and AOA within intertidal sediments. A study of ammonia oxidisers in North Sea sediments, characterised by low ammonia concentrations (3 to 53 µM) and dominated by AOA, reported the co-existence of metabolically active AOA and AOB. However, in the North Sea sediments *amoA* transcripts from AOB were more abundant than AOA, despite AOAs higher gene abundance^52^. On the other hand in Antarctic coastal waters AOA genes and transcripts were more abundant than AOB genes and transcripts^54^. Our data suggests that AOB are the primary drivers of AO in Rusheen bay, based on consistently higher spatial and temporal gene abundances, in addition to the higher *amoA* transcripts numbers, and significant correlation of AOB *amoA* genes and transcripts with PNR (Supplementary Table S3). While AOA *amoA* gene abundances also correlated significantly with PNR, AOA transcripts did not, nor with any other parameters (r = 0.515, P < 0.05; Supplementary Table S3).

Within Rusheen bay, the known *Nitrosomonas* group were the dominant AOB. The two most abundant AOB OTUs within the know *Nitrosomonas* (*amoA*bacD5 and D4) were present at both DNA and/or mRNA level and found at all sites. They are highly similar to *N*. *ureae* and clustered with similar phylotypes from estuarine environments characterised by high ammonia^55,56^. *N*. *ureae* and *N*. *oligotrophy*- like AOB can adapt to high initial ammonium concentrations (up to 10 mM) but also can grow at low ammonia concentrations (limiting concentration of 6 µM)^57^. This is consistent with ammonia concentrations within Rusheen bay where *in situ* concentrations never went above 1.2 mM NH_4_^+^.

The *Nitrosomonas* group A had low identity to known AOBs and were only recovered from muddy sites (Rsed_1, 24 psu), but T-RFLP data linked active phylotypes from this group across the entire bay. O’Mullan and Ward showed *Nitrosomonas* group A phylotypes associated with brackish water (15–30 psu)^58^. In contrast, sequences affiliated with *Nitrosomonas* group B were only present at the high salinity site and were closely related with sequences from marine coastal sediment sites with high salinity located in Monterey bay, California and Sendai bay, Japan, suggesting this group is associated with high salinity sites globally^58^.

AOA clustered with group I.1a. and were highly similar to Can. *Nitrosoarchaeum koreensis* MY1 at 99% similarity^59^ (Fig. 7). *N*. *koreensis* MY1, is inhibited by high salinity and grows optimally in 11 µM NH_4_^+^^60^. While OTUs from the MY1 cluster were present across the bay, transcripts were only recovered from the low salinity (0 psu), low ammonia (0.39 µM) site (RSed_6). Indeed, the majority of AOA *amoA* cDNA transcripts were recovered from this site, further, AOA gene abundance negatively correlated with high salinity over four time-points (Supplementary Table S2), indicating this cluster may be more active at low salinity. *Nitrosoarchaeum*-like sequences are consistently found in estuarine and freshwater sediments globally^61,62^ In San Francisco’s Sacramento-San Joaquin Delta this group was found in the low salinity sites where AOA tended to be more abundant than AOB^13,26^.

Overall, this study shows that AOB and AOA co-occur within intertidal sediments. AOB are likely a larger contributor to ammonia oxidation, based on their abundance at both DNA and mRNA level and through their significant correlations with PNR. However transcriptionally active AOA were also present. Further work to explore the co-occurrence and activity of AOA and AOB is needed. We also report evidence of small scale changes in ammonia oxidiser phylotypes across the bay selecting for AOB that are ubiquitously present and those that are only found at high salinity sites.

## Materials and Methods

### Site description and field sampling

Rusheen (53° 25.5894′N, −9° 11.9532′W) and Clew bay (53° 78.6962′N, −9° 64.9515′W), Ireland (Fig. 1) were sampled over four time-points: May, August, November 2013 and February 2014. Distances between sites ranged from ~200 m to 5 km apart within a site to 100 km apart between bays. At each site, at low tide, three replicates were collected from the top 2 cm of sediment, to ensure the top most layer of oxic sediment was sampled. Each replicate, composed of 10 random samples and was collected within a 10 m^2^ area, and stored on ice until return to the laboratory from which 0.5–0.7 g aliquots were flash frozen at −80 °C for molecular analysis. Samples for Potential Nitrification Rate (PNR) and physicochemical analysis were stored at 4 °C upon analysis. The Mastersizer 2000 Laser Particle Sizer (Malvern, UK) was used to determine particle sizes <1mm. Particles above 1 mm were analysed using a dry sieving technique^63^. Sediment was classified using the EUNIS A2 classification scheme. Ammonium (NH_4_^+^) and nitrate (NO_3_^−^) were extracted from the sediment with 1 M KCl and analysed using a colorimetric method^64^. Nitrite (NO_2_^−^) was extracted with 2 M KCl and analysed using Hach Nitriver 3 (Hach-Lange, Ireland). Total organic carbon (TOC) content was determined by combustion at 450 °C for 12 hours^63^.

### Potential nitrification rates (PNR)

Triplicate PNR containing 5 g sediment (wet-weight), 30 ml site-water amended with 24 µM sodium azide^65^ (NaN_3_, nitrite oxidation inhibitor) and (NH_4_)_2_SO_4_ at both 0 and 250 µM concentrations (Sigma Aldrich, Ireland), were incubated at 15 °C in the dark shaking (90 rpm) for 24 h. Nitrite was extracted and measured as described above. PNR rate was calculated as follows: $\frac{(mg/l\,Nitrite\times 60\,ml)}{(5\,g\,sediment)}$ = *PNR per g sediment per day*.

### DNA and RNA co-extraction

DNA and RNA was co-extracted from 0.5–0.7 g fresh sediment using a bead-beating protocol as detailed in Smith *et al*.^66^. Nucleic acids were re-suspended in 50 µl DEPC water of which a 25 µl aliquot was treated with TURBO DNase (Ambion, Ireland) according to the manufacturer’s instructions to prepare RNA. The remaining 25 µl aliquot was left untreated for use as DNA. The absence of DNA in the RNA fraction was confirmed by PCR of the 16 S *rRNA* (F63 and R518; Supplementary Table S5) using 2 µl RNA (neat to 10^−2^ dilution) as template. RNA was converted to cDNA using Superscript III (Life Science, USA) as in Smith *et al*., 2015, using gene specific primers targeting AOA or AOB *amo*A^3^. Each 10 µl RT reaction contained 8 µl of RNA, 2 mM of the appropriate reverse primer, ArchamoAR or BacamoA-2R (Supplementary Table S5), and 10 mM of each dNTP.

### *amoA* (RT)-Q-PCR

DNA standard curves were constructed from the target gene (Supplementary Table S5) according to Smith *et al*.^67^. A one-in-five dilution series of appropriate standard was used over an eight-point dynamic range from 10^10^ to 10^3^ gene copies per μl. *amoA* (Supplementary Table S5) was amplified from triplicate sediments collected from each site. Each 20 µl Q-PCR reaction mixture contained 10 µl EVA Green master mix (Biorad, Ireland), 0.4 µl of each primer (10 mM) (Supplementary Table S5), and 2 µl template DNA (10^−1^ dilution) or cDNA. Triplicate no-template controls (NTC) were included. Specificity of the amplicons was confirmed by melting curve analysis at the end of each (RT)-Q-PCR experimental run (Supplementary Table S5). The slope, y-intercept, and r^2^ values of standard curves are reported in Supplementary Table S6.

### Terminal restriction fragment length polymorphism (T-RFLP) of AOB *amoA* transcripts

AOB *amoA* cDNA from Ruhseen bay sites, February 2014 was amplified with a HEX labelled forward primer (Supplementary Table S5). Each 50 µl reaction mix contained 1 µl *amo*A AOB gene specific cDNA; 20 µM forward and reverse primers and 25 µl 2X My*Taq* mix (Bioline, Ireland) using a touchdown PCR (Supplementary Table S5). PCR products were purified using SureClean Plus (Bioline, Ireland) and re-suspended in 15 µl of sterile water. Five µl of PCR product was independently digested with three enzymes, *Taq*I, *Hha*I and *Alu*I, (Thermo Fisher Scientific, Ireland) according to manufacturer’s instructions. Samples were sent to Source Bioscience, Ireland, for T-RFLP analysis. Terminal Restriction Fragments (TRF) were sized against a ROX-genescan 500 internal size standard using Peakscanner software v1.0 (Applied Biosystems, Ireland). T-RFLP profiles per site from individual enzymes were combined into a single profile and aligned using T-Align^68^.

### Cloning and sequence analysis of bacterial and archaeal amoA genes and transcripts

AOA and AOB *amo*A genes and transcripts from replicate samples from sites RSed_1, RSed_5, RSed_6 & RSed_7 (Fig. 1) Rusheen Bay February 2014 were cloned and sequenced. 5 µl DNA (10^−1^) or cDNA (neat) was amplified as follows: 10 × 1.5 mM MgCl_2_ PCR buffer (Sigma Aldrich, Ireland), 0.2 mM of each dNTP (Sigma Aldrich, Ireland), 0.25 µM of each primer ArchamoAF, and ArchamoAR, or BacamoA1F and BacamoA-2R (Supplementary Table S5; Eurofins, UK), and 2.5 units of *Taq* polymerase (Sigma Aldrich, Ireland) using touchdown PCR (Supplementary Table S5). Amplicons were gel purified using a Gel Purification Kit (Qiagen, Ireland) according to manufacturer’s instructions and re-suspended in 15 µl sterile water. PCR products were cloned using PGem-T cloning kit (Promega, Ireland) according to manufacturer’s instructions. Fifty white colonies were screened using PGEM-T vector specific primers M13F and R (10 mM) (Supplementary Table S5). Clones containing inserts of the correct size were sent for sequencing using vector primers M13F/T7F (Source Bioscience, Ireland). *amoA* nucleotide sequences were translated into protein sequences using the Translate tool on ExPASy (Expert Protein Analysis System; http://us.expasy.org/tools/dna.html). Nucleotide and protein sequences were compared to entries in GenBank using BlastN and BlastP^69^. Protein sequence alignments were constructed using Bioedit (version 7.2)^70^. *amo*A gene sequences displaying more than 97% similarity were grouped into a single operational taxonomic unit (OTU) using DOTUR software by the furthest neighbour approach^71^. Distance matrices were calculated using the PROTDIST program in PHYLIP^72^. Phylogenetic trees were created from the distance matrices using the neighbour-joining method^73^ and Kimura substitution algorithm^74^ in MEGA-6^75^. Consensus trees were calculated after bootstrapping (1,000 replicate trees).

### Statistical analysis

All data was tested for normality using Kolmogorov-Smirnov test and log-transformed as necessary^76^. Variation in PNR, gene abundances and environmental variables between sites, bays and time-points were analysed using a three-way analysis of variance (ANOVA) followed by a post-hoc Bonferroni test^77^ in SPSS v21 (IBM, USA). A one-way ANOVA was used on environmental variables between sites in both bays at each time-point in SPSS v21. Analysis of covariance (ANCOVA) was conducted on gene abundance data by time-point in Graph Pad Prism v6 (GraphPad software, USA). A cluster dendrogram was created in Primer 7 (Quest research limited) using a bray-curtis similarity resemblance matrix, analysis of similarities (ANOSIM) was carried out between sites. Canonical correspondence analysis (CCA) using CANOCO 5 (http://www.canoco5.com) was used to explore relationships between community structure and environmental parameters. Explanatory value of environmental factors was determined using forward selection (tested by 499 Montecarlo permutations). Differences and correlation coefficients were considered significant at P < 0.05 unless otherwise stated in the text. Only significant explanatory variables were plotted.

### Data availability

The sequence datasets generated during and/or analysed during the current study are available in the NCBI repository, with the following accession numbers. AOA accession KX664728 - KX664820. AOB DNA accession numbers KX673291 - KX673360. AOB cDNA accession numbers KX690304 - KX690497. All other datasets generated during and/or analysed during the current study are available from the corresponding author on reasonable request.

## Electronic supplementary material

Supplementary Tables
