# Supplementary material for: Small-scale variation of ammonia oxidisers within intertidal sediments dominated by ammonia-oxidising bacteria Nitrosomonas sp. amoA genes and transcripts
Source: Sci Rep. 2017 Oct 16;7:13200. doi: 10.1038/s41598-017-13583-x (PMC5643298; doi:10.1038/s41598-017-13583-x)
Supplement: Supplementary file 1 — Supplementary Tables [file 41598_2017_13583_MOESM1_ESM.doc]

Small-scale variation of ammonia oxidisers within intertidal sediments dominated by ammonia-oxidising bacteria *Nitrosomonas sp. amoA* genes and transcripts

**Authors:** Aoife M. Duff^1^, Li-Mei Zhang^2^, Cindy J. Smith^1*^

Table S1A: Rusheen bay Environmental Parameters

| ***Time*** | **Site** | **NO_3_^-^ (µM) (± s.d.)** | **NO_2_^-^ (µM) (± s.d.)** | **NH_4_^+^ (µM) (± s.d.)** | **pH**  **(± s.d.)** | **TOC (%)**  **(± s.d.)** | **Sediment Temp (°C)** | **Salinity (psu)** | **Gravel (%)**  **(± s.d.)** | **Sand (%)**  **(± s.d.)** | **Mud (%)**  **(± s.d.)** | **Eunis Classification** |
| --- | --- | --- | --- | --- | --- | --- | --- | --- | --- | --- | --- | --- |
| *A*  *P*  *R*  *I*  *L* | **RSed_1** | 2.26  ± 0.78 | 0.33  ± 0.06 | 40.44  ± 16.39 | 7.48  ± 0.04 | 17.98  ± 1.4 | 11 | 32.08 | 0 | 42.2 ± 0.04 | 57.8 ± 0.04 | mS- muddy sand |
|  | **RSed_2** | 2.26  ± 0.78 | 0.22  ± 0.07 | 152.44  ± 68.32 | 7.7  ± 0.4 | 16.49  ± 0.4 | 11 | 32.08 | 2.0 ± 0.006 | 85.4 ±0.025 | 14.6 ± 0.025 | mS- muddy sand |
|  | **RSed_3** | 1.81  ± 0.78 | 0.1  ± 0.16 | 68.44  ± 17.68 | 7.79  ± 0.07 | 17.5  ± 1.6 | 11 | 32.08 | 0.1 ± 0.0003 | 98.5 ± 0.003 | 1.5 ± 0.003 | (m)S- slightly muddy sand |
|  | **RSed_4** | 1.80  ± 1.56 | 0.07  ± 0.095 | 12.44  ± 7.12 | 7.96  ± 0.03 | 12.57  ± 0.6 | 11 | 33.84 | 18.7 ± 0.05 | 66.4 ± 0.33 | 30.2 ± 0.3 | gmS - gravelly muddy sand |
|  | **RSed_5** | 0.90  ± 1.56 | 0.14  ± 0.045 | 203.78  ± 15 | 7.92  ± 0.02 | 17.8  ± 4.2 | 11 | 32.08 | 2.3 ± 0.01 | 94.7 ± 0.03 | 5.2 ± 0.03 | (g)mS- slightly gravelly muddy sand |
|  | **RSed_6** | 4.52  ± 2.82 | 0.14  ± 0.07 | 42  ± 9.33 | 7.71  ± 0.03 | 15.24  ± 1.05 | 11 | 0.92 | 3.4 ± 0.006 | 99.2 ± 0.005 | 0.8 ± 0.005 | (g)S- slightly gravelly sand |
|  | **RSed_7** | 0.90  ± 0.78 | 0.28  ± 0.1 | 113.56  ± 112.42 | 7.92  ± 0.008 | 17.14  ± 1.3 | 11 | 3.53 | 0.5 ± 0.003 | 99.4 ± 0.004 | 0.6 ± 0.004 | S- Sand |
| *A*  *U*  *G*  *U*  *S*  *T* | **RSed_1** | 2.26 ± 0.78 | 4.43  ± 0.52 | 141.56  ± 88.34 | 7.63  ±0.02 | 6.74  ± 1.06 | 22 | 16.66 | 11.6 ± 0.015 | 49.7 ± 0.02 | 38.7 ± 0.03 | gmS- gravelly muddy sand |
|  | **RSed_2** | 1.35 ± 2.74e-16 | 4.00  ± 0.40 | 96.44  ± 40.23 | 7.72  ± 0.01 | 2.06  ± 0.08 | 22 | 16.66 | 0.4 ± 0.002 | 84.7 ± 0.02 | 14.9 ± 0.02 | mS- muddy Sand |
|  | **RSed_3** | 1.80 ± 0.78 | 3.65  ± 0.26 | 85.56  ± 5.39 | 7.74  ± 0.01 | 2.65  ± 1.7 | 22 | 16.66 | 0.4 ± 0.002 | 97.9 ± 0.002 | 1.7 ± 0.002 | (m)S- slightly muddy sand |
|  | **RSed_4** | 0.451 ± 0.78 | 2.87  ± 0.45 | 48.22  ± 2.69 | 7.99  ± 0.05 | 6.97  ± 6.5 | 22 | 16.66 | 8.6 ± 0.03 | 91.3 ± 0.03 | 0.1 ± 0.0001 | gS - gravelly sand |
|  | **RSed_5** | 1.35 ± 2.74e-16 | 3.04 ± 0.66 | 96.44 ± 31.07 | 7.66 ± 0.03 | 1.46 ± 0.27 | 22 | 16.66 | 3.6 ± 0.03 | 89.4 ± 0.03 | 7 ± 0.02 | (g)mS- slightly gravelly muddy sand |
|  | **RSed_6** | 1.35 ± 2.74e-16 | 2.96 ± 0.6 | 73.11 ± 2.69 | 7.87 ± 0.04 | 0.96 ± 0.2 | 22 | 4.2 | 2.5 ± 0.008 | 97.3 ± 0.008 | 0.2 ± 0.0002 | (g)S- slightly gravelly sand |
|  | **RSed_7** | 3.16 ± 0.78 | 0.17 ± 0.3 | 304.89 ± 2.69 | 7.87 ± 0.03 | 0.83 ± 0.2 | 22 | 20.05 | 0.3 ± 0.001 | 99.5 ± 0.001 | 0.2 ± 0.0005 | S- Sand |
| *N*  *O*  *V*  *E*  *M*  *B*  *E*  *R* | **RSed_1** | 3.42 ± 3.88 | 2.87 ± 0.26 | 894.42 ± 120.84 | 7.95 ± 0.05 | 15.02 ± 8.3 | 8 | 32 | 56.4 ± 0.12 | 24.3 ± 0.05 | 19.3 ± 0.07 | msG- muddy sandy gravel |
|  | **RSed_2** | 4.69 ± 3.72 | 2.87 ± 0.9 | 843.91 ± 49.63 | 8.13 ± 0.04 | 2.65 ± 0.29 | 8 | 32 | 2.1 ± 0.009 | 87.1 ± 0.02 | 10.7 ± 0.008 | (g)mS- slightly gravelly muddy Sand |
|  | **RSed_3** | 3.31 ± 2.3 | 3.04 ± 0.15 | 1107.6 ± 37.96 | 8.14 ± 0.08 | 1.5 ± 0.07 | 8 | 32 | 3.2 ± 0.002 | 96.6 ± 0.002 | 0 | (g)S- slightly gravelly sand |
|  | **RSed_4** | 0.61 ± 1.07 | 2.43 ± 1.34 | 689.04 ± 12.98 | 8.27 ± 0.5 | 2.14 ± 0.67 | 8 | 32 | 8.9 ± 0.042 | 85.7 ± 0.04 | 5.4 ± 0.01 | gmS - gravelly muddy sand |
|  | **RSed_5** | 0.98 ± 0.98 | 3.04 ± 0.54 | 1166.20 ± 80.86 | 7.91 ± 0.02 | 1.75 ± 0.08 | 8 | 32 | 6.3 ± 0.02 | 90.4 ± 0.016 | 3.3 ± 0.004 | (m)gS- slightly muddy gravelly sand |
|  | **RSed_6** | 2.91 ± 3.72 | 4.17 ± 1.14 | 885.21 ± 123.96 | 7.94 ± 0.02 | 1.06 ± 0.1 | 8 | 20 | 0.2 ± 0.0007 | 99.7 ± 0.0006 | 0.1 ± 0.0008 | S - Sand |
|  | **RSed_7** | 1.06 ± 1.64 | 2.78 ± 0.4 | 781.4 ± 37.4 | 8.13 ± 0.02 | 0.69 ± 0.4 | 8 | 10 | 1.3 ± 0.003 | 98.7 ± 0.003 | 0 | (g)S- slightly gravelly sand |
| *F*  *E*  *B*  *R*  *U*  *A*  *R*  *Y* | **RSed_1** | 3.21 ± 0.37 | 1.13 ± 1.96 | 0 ± 0 | 7.8 ± 0.04 | 8.43 ± 2.2 | 10 | 24 | 37.5 ± 0.08 | 44.5 ± 0.08 | 17.9 ± 0.003 | mgS- muddy gravelly sand |
|  | **RSed_2** | 2.18 ± 0.42 | 3.91 ± 5.9 | 19.94 ± 34.54 | 7.88 ± 0.03 | 3.78 ± 0.02 | 10 | 24 | 1.3 ± 0.003 | 77.4 ± 0.02 | 21.4 ± 0.014 | (g)mS- slightly gravelly muddy Sand |
|  | **RSed_3** | 2.53 ± 0.49 | 0.61 ± 1.05 | 15.44 ± 13.39 | 7.78 ± 0.05 | 1.33 ± 0.06 | 10 | 24 | 0 | 99.8 ± 0.001 | 0.2 ± 0.001 | S- sand |
|  | **RSed_4** | 0.37 ± 0.14 | 0 ± 0 | 0 ± 0 | 7.15 ± 0.1 | 1.16 ± 0.02 | 10 | 24 | 0.8 ± 0.007 | 99.2 ± 0.008 | 0 | S- Sand |
|  | **RSed_5** | 3.92 ± 1.71 | 0.61 ± 0.84 | **56.56 ±** 57.26 | 7.62 ± 0.04 | 1.49 ± 0.14 | 10 | 24 | 0.1 ± 0.0002 | 95.4 ± 0.005 | 4.5 ± 0.005 | (m)S- slightly muddy sand |
|  | **RSed_6** | 7.69 ± 7.08 | 0.43 ± 0.75 | 0.39 ± | 6.65 ± 0.14 | 1.59 ± 0.16 | 10 | 0 | 1.6 ± 0.005 | 98.4 ± 0.005 | 0 | (g)S- slightly gravelly sand |
|  | **RSed_7** | 3.18 ± 1.13 | 0 ± 0 | 0 ± 0 | 7.99 ± 0.02 | 0.94 ± 0.04 | 10 | 9 | 0.2 ± 0.0009 | 99.8 ± 0.0009 | 0 | S- Sand |
|  |  | | | | | | | | | | | |

Table S1B: Clew bay Environmental Parameters

| ***Time*** | **Site** | **NO_3_^-^ (µM) (± s.d.)** | **NO_2_^-^ (µM) (± s.d.)** | **NH_4_^+^ (µM) (± s.d.)** | **pH**  **(± s.d.)** | **TOC (%)**  **(± s.d.)** | **Sediment Temp (°C)** | **Salinity (psu)** | **Gravel (%)**  **(± s.d.)** | **Sand (%)**  **(± s.d.)** | **Mud (%)**  **(± s.d.)** | **Eunis Classification** |
| --- | --- | --- | --- | --- | --- | --- | --- | --- | --- | --- | --- | --- |
| *A*  *P*  *R*  *I*  *L* | **CSed_1** | 0.45 ± 0.78 | 2.96 ± 0.4 | 269.11 ± 31.07 | 7.53 ± 0.02 | 20.3 ± 0.23 | 11 | 32.13 | 0 | 48.3 ± 0.02 | 51.7 ± 0.02 | sM- sandy mud |
|  | **CSed_2** | 1.35 ± 1.35 | 3.13 ± 0.26 | 45.11 ± 16.39 | 7.60 ± 0.2 | 14 ± 0.8 | 11 | 30.69 | 0.2± 0.00016 | 97.3 ± 0.006 | 2.7 ± 0.006 | (m)S- slightly muddy sand |
|  | **CSed_3** | 0.45 ± 0.78 | 2.43 ± 0.15 | 7.78 ± 9.71 | 8.18 ± 0.04 | 14.59 ± 0.8 | 11 | 32 | 0.3 ± 0.0015 | 99.8 ± 0.0003 | 0.2 ± 0.0003 | S- Sand |
|  | **CSed_4** | No Sampling was carried out at this time point | | | | | | | | | | |
|  | **CSed_5** | 1.806 ± 0.78 | 2.52 ± 0.66 | 126 ± 36.44 | 7.56 ± 0.2 | 13.38 ± 1.99 | 11 | 33.41 | 0 | 55.4 ± 0.009 | 44.6 ± 0.009 | mS- muddy sand |
|  | **CSed_6** | 1.80 ± 0.78 | 3.91 ± 0.26 | 122.89 ± 9.71 | 7.67 ± 0.02 | 15.88 ± 1.85 | 11 | 0.23 | 12.8 ± 0.01 | 89.3 ± 0.007 | 10.6 ± 0.007 | gmS - gravelly muddy sand |
| *A*  *U*  *G*  *U*  *S*  *T* | **CSed_1** | 1.35 ± 2.74e-16 | 2.35 ± 0 | 182 ± 51.33 | 7.87 ± 0.03 | 4.34 ± 0.2 | 22 | 34.35 | 1.2 ± 0.008 | 41.7 ± 0.018 | 57.2 ± 0.02 | (g)sM- slightly gravelly sandy mud |
|  | **CSed_2** | 1.35 ± 2.74e-16 | 2.43 ± 0.4 | 56 ± 0 | 7.8 ± 0.16 | 0.78 ± 0.12 | 22 | 34.35 | 0.1 ± 0.0001 | 98.9 ± 0.006 | 0.4 ± 0.0008 | S- Sand |
|  | **CSed_3** | 0.90 ± 0.78 | 2.35 ± 0 | 62.22 ± 2.69 | 8.13 ± 0.03 | 0.87 ± 0.04 | 22 | 34.35 | 0.7 ± 0.0009 | 99.2 ± 0.001 | 0.1 ± 0.0002 | S- Sand |
|  | **CSed_4** | 1.35 ± 0 | 2.26 ± 0.91 | 68.44 ± 9.71 | 8.26 ± 0.015 | 0.64 ± 0.06 | 22 | 34.35 | 0.3 ± 0.001 | 99.3 ± 0.0005 | 0.4 ± 0.0005 | S- Sand |
|  | **CSed_5** | 3.16 ± 3.13 | 2.35 ± 0.52 | 158.67 ± 20.34 | 7.82 ± 0.03 | 2.58 ± 0.38 | 22 | 34.35 | 0.8 ± 0.004 | 60.2 ± 0.03 | 39 ± 0.03 | mS- muddy sand |
|  | **CSed_6** | 1.80 ± 0.78 | 3.83 ± 0.8 | 108.89 ± 15 | 7.83 ± 0.02 | 1.45 ± 0.14 | 22 | 0.07 | 31.4 ± 0.03 | 62.9 ± 0.03 | 5.7 ± 0.003 | gmS - gravelly muddy sand |
| *N*  *O*  *V*  *E*  *M*  *B*  *E*  *R* | **CSed_1** | 0.6 ± 0.52 | 0 | 45.48 ± 55.38 | 7.68 ± 0.04 | 4.78 ± 0.67 | 9 | 36 | 27.5 ± 0.03 | 36.4 ± 0.05 | 36.1 ± 0.02 | gmS- gravelly muddy sand |
|  | **CSed_2** | 0.67 ± 0.71 | 0.522 ± 0.69 | 0 ± 0 | 8.03 ± 0.06 | 0.5 ± 0.09 | 9 | 36 | 0.3 ± 8.02e-5 | 99.5 ± 0.002 | 0.2 ± 0.0016 | S- Sand |
|  | **CSed_3** | 0.43 ± 0.76 | 0.96 ± 1.53 | 0 ± 0 | 7.89 ± 0.1 | 0.98 ± 0.2 | 9 | 36 | 1.8 ± 0.005 | 98.2 ± 0.005 | 0 | (g)S- slightly gravelly sand |
|  | **CSed_4** | 1.07 ± 1.54 | 1.04 ± 1.3 | 0 ± 0 | 8.03 ± 0.07 | 0.74 ± 0.03 | 9 | 36 | 0.5 ± 0.001 | 99.5 ± 0.001 | 0 | S- Sand |
|  | **CSed_5** | 0.56 ± 0.13 | 1.22 ± 0.92 | 95.15± 25 | 7.76 ± 0.03 | 3.18 ± 0.83 | 9 | 36 | 19.8 ± 0.03 | 61.4 ± 0.08 | 18.7 ± 0.04 | gmS - gravelly muddy sand |
|  | **CSed_6** | 1.58 ± 0.68 | 1.65 ± 2.64 | 0 ± 0 | 7.96 ± 0.04 | 2.74 ± 0.9 | 9 | 0 | 55.9 ± 0.03 | 38.9 ± 0.02 | 5.1 ± 0.008 | msG- muddy sandy gravel |
| *F*  *E*  *B*  *R*  *U*  *A*  *R*  *Y* | **CSed_1** | 0 ± 0 | 1.57 ± 0.45 | 0 ± 0 | 7.85 ± 0.03 | 13.68 ± 4 | 8 | 34 | 22 ± 0.08 | 52.6 ± 0.07 | 25.4 ± 0.03 | gmS- gravelly muddy sand |
|  | **CSed_2** | 0.1 ± 0.01 | 0.87 ± 0.6 | 0 ± 0 | 8.28 ± 0.042 | 0.54 ± 0.18 | 8 | 34 | 0.1 ± 0.0005 | 99.9 ± 0.0005 | 0 | S- Sand |
|  | **CSed_3** | 0.48 ± 0.84 | 1.22 ± 0.54 | 0 ± 0 | 7.75 ± 0.05 | 0.58 ± 0.09 | 8 | 34 | 2.2 ± 0.02 | 97.8 ± 0.02 | 0 | (g)S- slightly gravelly sand |
|  | **CSed_4** | 0.42 ± 0.73 | 4.43 ± 1.59 | 0 ± 0 | 8.43 ± 0.07 | 0.76 ± 0.03 | 8 | 34 | 0.1 ± 0.0003 | 98.1 ± 0.009 | 1.8 ± 0.009 | (m)S- slightly muddy sand |
|  | **CSed_5** | 0 ± 0 | 2.35 ± 0.78 | 182.44 ± 29.84 | 8.35 ± 0.02 | 12.99 ± 8.5 | 8 | 34 | 0.2 ± 0.0009 | 26.4 ± 0.05 | 73.4 ± 0.05 | sM- sandy mud |
|  | **CSed_6** | 0.58 ± 0.34 | 1.39 ± 0.66 | 0 ± 0 | 8.05 ± 0.1 | 1.56 ± 0.34 | 8 | 0 | 10.7 ± 0.05 | 78.4 ± 0.07 | 10.9 ± 0.03 | gmS - gravelly muddy sand |
|  |  | | | | | | | | | | | |

**Table S2: Significant Spearmann Rank Correlations carried out on all time-points in Rusheen bay dataset (temporally)**

|  |  | **PNR** | **TOC** | **Salinity** | **pH** | **Sediment Temp** | **NH_4_^+^** |
| --- | --- | --- | --- | --- | --- | --- | --- |
| **PNR** | Correlation Coefficient | - | 0.223 | 0.265 | - | - | - |
|  | Significance (2-tailed) | - | 0.041 | 0.015 | - | - | - |
|  | N | - | 84 | 84 | - | - | - |
| **AOA**  **gene abundance** | Correlation Coefficient | - | - | -0.245 | 0.69 | 0.242 | -0.438 |
|  | Significance (2-tailed) | - | - | 0.025 | 0.000 | 0.026 | 0.000 |
|  | N | - | - | 84 | 84 | 84 | 84 |
| **AOB**  **gene abundance** | Correlation Coefficient | 0.509 | 0.434 | - | 0.315 | -0.459 | -0.404 |
|  | Significance (2-tailed) | 0.000 | 0.000 | - | 0.004 | 0.000 | 0.000 |
|  | N | 84 | 84 | - | 84 | 84 | 84 |

Transcript abundance was not completed for all time-points and so is not included here

Table S3: Significant Spearmann Rank Correlations carried out on February 2014 dataset (spatially)

|  |  | **PNR** | **TOC** | **Sediment Salinity** | **pH** | **NO_3_^-^** |
| --- | --- | --- | --- | --- | --- | --- |
| **PNR** | Correlation Coefficient | - | 0.651 | 0.573 | 0.530 | - |
|  | Significance (2-tailed) | - | 0.001 | 0.007 | 0.014 | - |
|  | N | - | 21 | 21 | 21 | - |
| **AOB**  **gene abundance** | Correlation Coefficient | 0.873 | 0.697 | 0.677 | - | - |
|  | Significance (2-tailed) | 0.000 | 0.000 | 0.001 | - | - |
|  | N | 21 | 21 | 21 | - | - |
| **AOA**  **gene abundance** | Correlation Coefficient | 0.515 | - | 0.463 | - | - |
|  | Significance (2-tailed) | 0.017 | - | 0.034 | - | - |
|  | N | 21 | - | 21 | - | - |
| **AOB transcript abundance** | Correlation Coefficient | 0.868 | 0.553 | - | - | 0.444 |
|  | Significance (2-tailed) | 0.000 | 0.009 | - | - | 0.044 |
|  | N | 21 | 21 | - | - | 21 |

Table S4: Clone library values

|  | **DNA** | | | | **cDNA** | | | |
| --- | --- | --- | --- | --- | --- | --- | --- | --- |
| **Samples** | **AOB** | | **AOA** | | **AOB** | | **AOA** | |
|  | **No. of Clones** | **No. of OTUs** | **No. of Clones** | **No. of OTUs** | **No. of Clones** | **No. of OTUs** | **No. of Clones** | **No. of OTUs** |
| **Rsed_1** | 24 | 15 | 31 | 9 | 40 | 26 | 0 | 0 |
| **Rsed_5** | 24 | 12 | 9 | 6 | 60 | 5 | 3 | 3 |
| **Rsed_6** | 20 | 8 | 21 | 4 | 38 | 7 | 29 | 5 |
| **Rsed_7** | 2 | 1 | 0 | 0 | 56 | 3 | 0 | 0 |
| **Total** | 70 | 36 | 61 | 19 | 194 | 41 | 32 | 8 |

**Table S5:** Suite of primers used in this study

| Primer Name | Primer sequence (5′-3′) | Target gene | Thermal Profile | Reference |
| --- | --- | --- | --- | --- |
| F63 | CAG GCC TAA CAC ATG GCA AGT C | bacterial 16S rRNA gene | 95 °C, 5 min; 30 x( 95°C, 30 s; 57°C, 30 s; 72°C, 1 min) 72 °C, 7 min | (van Marchesi et al., 1998; Muyzer et al., 1993) |
| R518 | ATT ACC GCG GCT GCT GG |  |  |  |
| Arch-*amo*AF | STA ATG GTC TGG CTT AGA CG | archaeal *amoA* gene (635 bp) | Touchdown- 94°C, 3 min; 10X (94°C, 30 s; 62°C (AOB)/60°C (AOA), 45 s; -0.5°C at 3 °/s; 72°C, 1 min) 30X (94°C, 30s; 57°C (AOB)/55°C (AOA), 45s; 72°C, 1 min) 72°C, 10 min  Q-PCR- 95°C, 3min; 40×(95°C, 30s; 55°C(Bac)/ 53°C (Arch) 30s; 72°C, 1 min, 81°C with plate read); Melt curve 65°C to 95°C, increment 0.5°C, 0:05+ plate read | (Franci*s et* al., 2005) |
| Arch-*amo*AR | GCG GCC ATC CAT CTG TAT GT |  |  |  |
| HEX-BacamoA-1F | GGG GTT TCT ACT GGT GGT |  |  |  |
| BacamoA-1F | GGG GTT TCT ACT GGT GGT | bacterial *amoA* gene (435 bp) |  | (Rotthauw*e et al*., 1997) |
| BacamoA-2R | CCC CTC KGS AAA GCC TTC TTC |  |  |  |
| M13F | TGT AAA ACG ACG GCC AGT | vector primers | 95°C, 5min; 30×(95°C, 30s; 57°C, 30s; 72°C, 1 min) 72°C,10min | (Invitrogen, Ireland) |
| T7F | TAA TAC GAC TCA CTA TAG GG |  |  |  |
| M13R | CAG GAA ACA GCT ATG AC |  |  |  |

Wobbles included: S: G/C; K: G/T; Y: C/T

**Table S6**: qPCR standard curve descriptors

| **Nucleic Acid** | **Genetic Target** |  | **Std. Curve Descriptions** | |  |  |
| --- | --- | --- | --- | --- | --- | --- |
|  |  | **Slope** | **% Efficiency** | **Y Intercept** | **R^2^** | **NTC** |
| DNA | Archaea *amo*A | 3.60 | 89.5 | 38.99 | 0.99 | 32.5 |
| DNA | Bacteria *amo*A | 3.52 | 92.5 | 37.68 | 0.99 | 30.75 |
| cDNA | Archaea *amo*A | 3.59 | 90 | 37.75 | 1 | 35 |
| cDNA | Bacteria *amo*A | 3.31 | 100.5 | 37.25 | 0.99 | 30 |
